# *MIR31HG* polymorphisms are related to steroid-induced osteonecrosis of femoral head among Chinese Han population

**DOI:** 10.1186/s12891-022-05785-w

**Published:** 2022-09-03

**Authors:** Yuan Wang, Yexin Wang, Da Liang, Hongtao Hu, Guangwei Li, Xiaoguang Meng, Bing Zhu, Wei Zhong

**Affiliations:** 1 Department of Joint Surgery, Affiliated Hospital of Weifang Medical University, #2428 Yuhe Road, Weifang, 261031 Shandong China; 2 Department of Spine Surgery, Affiliated Hospital of Weifang Medical University, Weifang, Shandong 261031 China

**Keywords:** *MIR31HG*, Osteonecrosis, Polymorphism, Steroid, Gene

## Abstract

**Backgrounds:**

*MIR31* host gene (*MIR31HG*) polymorphisms play important roles in the occurrence of osteonecrosis. However, the association of *MIR31HG* polymorphisms with the risk of steroid-induced osteonecrosis of the femoral head (SONFH) remains unclear. In this study, we aimed to investigate the correlation between *MIR31HG* polymorphisms and SONFH susceptibility in the Chinese Han population.

**Methods:**

A total of 708 volunteers were recruited to detect the effect of seven single nucleotide polymorphisms (SNPs) in the MIR31HG gene on SONFH risk in the Chinese Han population. Genotyping of MIR31HG polymorphisms was performed using the Agena MassARRAY platform. The odds ratio (OR) and 95% confidence interval (95% CI) were used to evaluate the correlation between MIR31HG polymorphisms and SONFH risk using logistic regression model.

**Results:**

According to the results of genetic model, rs10965059 in *MIR31HG* was significantly correlated with the susceptibility to SONFH (OR = 0.56, *p* = 0.002). Interestingly, the stratified analysis showed that rs10965059 was associated with the reduced risk of SONFH in subjects aged > 40 years (OR = 0.30, *p* < 0.001) and male populations (OR = 0.35, *p* < 0 .001). Moreover, rs10965059 was associated with the reduced risk of bilateral SONFH (OR = 0.50, *p* = 0.002). Finally, multi-factor dimension reduction (MDR) results showed that the combination of rs1332184, rs72703442, rs2025327, rs55683539, rs2181559, rs10965059 and rs10965064 was the best model for predicting SONFH occurrence (*p* < 0.0001).

**Conclusion:**

The study indicated that rs10965059 could be involved in SONFH occurrence in the Chinese Han population, which might provide clues for investigating the role of *MIR31HG* in the pathogenesis of SONFH.

**Supplementary Information:**

The online version contains supplementary material available at 10.1186/s12891-022-05785-w.

## Introduction

Osteonecrosis of the femoral head (ONFH) is a progressive rupture of the femoral head caused by the death of bone cells from various causes. The main characteristics of ONFH are the differentiation and the damage of bone marrow mesenchymal cells, enhanced cytotoxicity and destruction of vascular blood flow [1]. Osteonecrosis can be summarized into two categories: traumatic and non-traumatic femoral head necrosis. The steroid-induced osteonecrosis of the femoral head (SONFH), a non-traumatic femoral head necrosis, is a devastating disease, which is often result in devastating and crippling health conditions following steroid therapy [2]. In China, there were approximately 8 million patients with non-traumatic ONFH, which may be closely related to their frequent use of high-dose hormone therapy [3]. The pathogenesis is likely multifactorial, with genetic and environmental factors playing a role. Corticosteroid use, alcohol consumption, smoking, and infection and metabolic disease are all risk factors for SONFH [4]. Furthermore, genetics appears to play an important role in the development of SONFH. Previous studies have suggested that some genes play a role in SONFH occurrence including eNOS, PAI-1, VEGF, and ApoA [5]. However, there are still a large number of potential osteonecrosis-related genes and loci that have not been fully explored.

Long non-coding RNA (LncRNA) is a non-protein coding RNA molecule with a structure size of 200 nucleotides [6, 7]. Yuan and Sun’s studies showed that, lncRNA can regulate the development of immune diseases and affect immune function and autoimmunity, such as osteosarcoma and IgA nephropathy (IgAN) [8, 9]. The lncRNA *MIR31* host gene (*MIR31HG*) is an crucial regulator of malignant tumors [10]. *MIR31HG* located on chromosome 9 with the length of 2166 bp, is an lncRNA that acts on the progression of cancers, such as osteosarcoma, lung cancer, breast cancer and cervical cancer [9, 11, 12]. For example, recent studies have mentioned that *MIR31HG* is also involved in the development and regeneration of bone, and the pathogenesis of numerous orthopaedic conditions [13]. *MIR31HG* was up-regulated in osteosarcoma (OS) tissues and OS cell lines. In the case of bone loss, it was usually inflamed in the defective or injured tissue. A previous research has demonstrated that knock-down of *MIR31HG* not only affects the enhancement of osteogenic differentiation, but also limits the inhibitory effect of osteogenic in an inflammatory environment [14]. Studies have found that the interference with *MIR31HG* can improve osteogenesis in bone marrow stromal cells in patients with cleidocranial dysplasia (BMSCs-CCD), possibly through by promoting osteogenic differentiation and improving the aging-related properties of BMSCs-CCD [15]. *MIR31HG* can regulate the tumor suppressor miR-361 and its target genes, and promote tumor progression in osteosarcoma acting as an oncogene [9]. These studies have shown that *MIR31HG* may play an important role in SONFH. At present, the connection between *MIR31HG* gene polymorphism and the susceptibility to SONFH was not reported.

In the case-control study, the MassARRAY platform was used to select seven single nucleotide polymorphisms (SNPs) in *MIR31HG* for genotyping. We further investigated the effect of *MIR31HG* genetic polymorphisms on SONFH risk and conducted the stratified analysis to identify the contribution of confounding factors to the association between SNPs and the risk of SONFH. Our research will provide a new perspective to study the role of *MIR31HG* on the susceptibility to SNOFH.

## Methods

### Subjects

A total of 708 unrelated participants were recruited, embracing 200 SONFH cases (41.15 ± 12.90 years) and 508 healthy controls (42.70 ± 13.01 years) geographically and ethnically matched. Exclusion criteria were as follows: (1) Patients who did not meet the diagnostic criteria of SONFH and patients with traumatic ONFH, hip dislocation and other hip diseases; and (2) Patients without major family genetic diseases. The histopathological diagnosis was based on X-rays and/or magnetic resonance imaging (MRI) examination of the hip and frog positions. The research protocol was in compliance with the Declaration of Helsinki and was approved by the ethics committee of Affiliated Hospital of Weifang Medical University and Second Affiliated Hospital of Inner Mongolia Medical University. All experimental subjects signed a written informed consent. Demographic and blood biochemical indicators of each subject were collected from standardized questionnaires and medical records by trained research staff.

### Selection and genotyping for *MIR31HG* polymorphisms

Seven functional SNPs in *MIR31HG* (rs1332184, rs72703442, rs2025327, rs55683539, rs2181559, rs10965059 and rs10965064) were selected from the 1000 Genomes Project (http://www.1000genomes.org/), with the minor allele frequency (MAF) of each SNP greater than 0.05. Peripheral blood genomic DNA was extracted from all subjects according to the operating procedures of Whole Blood Genomic DNA Isolation Kit (Xi’an GoldMag Biotechnology, China). Agena MassARRAY iPLEX platform was used for genotyping. Agena Bioscience Assay was used to design PCR primers for amplification (Supplementary Table 1). Finally, Agena Bioscience TYPER application software 4.0 was performed to analyze the genetic data.

### Statistical analysis

The differences in demographic or clinical characteristics between the case and the control groups were compared by χ^2^tests for the categorical variables and Student’s t-tests for continuous variables. PLINK software was used to detect four genetic models (co-dominant, dominant, recessive, and log-additive). Hardy-Weinberg equilibrium (HWE) of all SNPs from control individuals was evaluated by χ^2^ test. Multi-factor dimension reduction (MDR) is suitable for detecting the interaction between SNP-SNP and SONFH risk. Analysis of variance (ANOVA) was performed to determine differences in clinical characteristics among SNPs genotypes. By calculating the odds ratio (OR) and 95% confidence interval (CI), logistic regression results were adjusted for age and gender to assess the impact of *MIR31HG* polymorphism on SONFH risk. HaploView software version 4.2 and logistic regression were carried out to assess the correlation of *MIR31HG* haplotypes with SONFH susceptibility. All statistics were two-tailed, and a *p* < 0.05 was considered statistically significant. SPSS 20.0 software (Chicago, IL, USA) was used for statistical analysis in this study.

## Results

### Basic conventional characteristics

were consisted. The basic characteristics of 200 patients with SONFH and 508 healthy participants are summarized in Table 1, including age, gender, necrosis, and course. The mean age was 41.15 ± 12.90 years in the case group and 42.70 ± 13.01 years in control group. There were no significant differences in age (*p* = 0.152) and gender (*p* = 0.706) characteristics between cases and controls.

**Table 1:** Basic characteristic of SONFH patients and healthy subjects in this study

Characteristics	Cases (*n* = 200)	Controls (*n* = 508)	*p*
Age, years (mean ± SD)	41.15 ± 12.90	42.70 ± 13.01	0.152^a^
> 40	100 (50%)	290 (57%)	
≤ 40	100 (50%)	218 (43%)	0.706^b^
Gender			
Male	117 (59%)	425 (84%)	
Female	83 (41%)	83 (16%)	
Necrosis			
Bilateral	143 (72%)		
Missing	55 (28%)		
Course (months)			
> 29	61 (31%)		
≤ 29	139 (69%)		

*SD* Standard deviation

*p*^a^ values were calculated from student’s t test

*p*^b^ values were calculated from χ^2^ test

### Association analysis of *MIR31HG* and SONFH risk

In this study, seven SNPs (rs1332184, rs72703442, rs2025327, rs55683539, rs2181559, rs10965059, and rs10965064) were successfully genotyped. The minor allele frequencies are record in Table 2. All SNP distribution of controls were in line with HWE (*p* > 0.05). The rs10965059-T allele frequency in the case group (0.103) was lower than that in the control group (0.169), and the reduced risk of SONFH was found (OR = 0.56, *p* = 0.002).

**Table 2:** Basic information for *MIR31HG* SNPs

SNP ID	Chromosome position	Role	Alleles A/B	MAF		O (HET)	E (HET)	*P*^a^-HWE	OR (95% CI)	*P*^b^
				Case	control					
rs1332184	chr9:21504203	Intron	A/G	0.245	0.264	0.383	0.389	0.732	0.90 (0.69–1.18)	0.456
rs72703442	chr9:21515795	Intron	A/C	0.143	0.163	0.286	0.273	0.328	0.86 (0.62–1.19)	0.347
rs2025327	chr9:21531629	Intron	C/T	0.108	0.122	0.217	0.214	0.999	0.87 (0.60–1.25)	0.445
rs55683539	chr9:21542134	Intron	T/C	0.230	0.244	0.341	0.369	0.093	0.92 (0.71–1.22)	0.590
rs2181559	chr9:21543938	Intron	A/T	0.332	0.359	0.443	0.460	0.387	0.89 (0.69–1.13)	0.328
rs10965059	chr9:21544062	Intron	T/C	0.103	0.169	0.299	0.281	0.204	0.56 (0.39–0.81)	0.002*
rs10965064	chr9:21553538	Intron	G/C	0.358	0.370	0.461	0.466	0.776	0.94 (0.74–1.21)	0.658

*SNP* Single nucleotide polymorphism, *MAF* Minor allele frequency, *HWE* Hardy-Weinberg equilibrium

*P*^a^ -values were calculated by exact test *P*^a^ < 0.05 are excluded

*P*^*b*^ -values were calculated by two-sided χ^2^. *P*^b^ < 0.05 indicates statistical significance

* indicates statistical strongly significance (*p* < 0.01)

The correlation between the risk of SONFH and the *MIR31HG* polymorphisms was assessed after adjusting for age and gender in the four genetic models (co-dominant, dominant, recessive, and log-addition models). Our analysis results showed that rs10965059 was significantly related to the risk of SONFH (Table 3). In addition, rs10965059 was associated with the reduced susceptibility to SONFH in the co-dominant (T/C vs. C/C, OR = 0.50, *p* = 0.002), dominant (T/T-T/C vs. C/C, OR = 0.54, *p* = 0.004) and log-additive (OR = 0.63, *p* = 0.016) models.

**Table 3:** Association analysis between *MIR31HG* SNPs and SONFH risk

SNP ID	Model	Genotype	Case	Control	With Adjustment	
					OR (95% CI)	*P*^*b*^
rs1332184	Codominant	G/G	109	276	1	
		A/A	7	37	0.49 (0.20–1.14)	0.099
		A/G	84	194	1.05 (0.74–1.50)	0.770
	Dominant	G/G	109	276	1	
		A/A-A/G	91	231	0.96 (0.68–1.36)	0.837
	Recessive	A/G-G/G	193	470	1	
		A/A	7	37	0.47 (0.20–1.11)	0.084
	Log-additive	–	–	–	0.88 (0.67–1.17)	0.391
rs72703442	Codominant	C/C	145	352	1	
		A/A	2	10	0.52 (0.11–2.49)	0.414
		A/C	53	145	0.85 (0.58–1.25)	0.417
	Dominant	C/C	145	352	1	
		A/A-A/C	55	155	0.83 (0.57–1.22)	0.342
	Recessive	A/C-C/C	198	497	1	
		A/A	2	10	0.54 (0.11–2.59)	0.446
	Log-additive	–	–	–	0.82 (0.58–1.17)	0.285
rs2025327	Codominant	T/T	159	391	1	
		C/C	2	7	0.61 (0.12–3.13)	0.557
		C/T	39	110	0.84 (0.55–1.28)	0.407
	Dominant	T/T	159	391	1	
		C/C-C/T	41	117	0.82 (0.54–1.25)	0.354
	Recessive	C/T-T/T	198	501	1	
		C/C	2	7	0.64 (0.13–3.24)	0.588
	Log-additive	–	–	–	0.82 (0.56–1.21)	0.324
rs55683539	Codominant	C/C	116	297	1	
		T/T	8	37	0.57 (0.25–1.29)	0.176
		T/C	76	173	1.12 (0.78–1.60)	0.536
	Dominant	C/C	116	297	1	
		T/T-T/C	84	210	1.02 (0.73–1.45)	0.893
	Recessive	T/C-C/C	192	470	1	
		T/T	8	37	0.54 (0.24–1.22)	0.139
	Log-additive	–	–	–	0.93 (0.71–1.24)	0.629
rs2181559	Codominant	T/T	87	213	1	
		A/A	20	70	0.64 (0.36–1.15)	0.135
		A/T	92	225	1.05 (0.73–1.50)	0.804
	Dominant	T/T	87	213	1	
		A/A-A/T	112	295	0.95 (0.67–1.33)	0.746
	Recessive	A/T-T/T	179	438	1	
		A/A	20	70	0.63 (0.36–1.09)	0.097
	Log-additive	–	–	–	0.87 (0.68–1.12)	0.292
rs10965059	Codominant	C/C	164	340	1	
		T/T	5	10	1.12 (0.36–3.49)	0.842
		T/C	31	149	0.50 (0.32–0.78)	0.002*
	Dominant	C/C	164	340	1	
		T/T-T/C	36	159	0.54 (0.35–0.82)	0.004*
	Recessive	T/C-C/C	195	489	1	
		T/T	5	10	1.31 (0.42–4.07)	0.644
	Log-additive	–	–	–	0.63 (0.43–0.92)	0.016*
rs10965064	Codominant	C/C	80	203	1	
		G/G	23	71	0.82 (0.47–1.42)	0.474
		G/C	97	234	1.00 (0.70–1.45)	0.989
	Dominant	C/C	80	203	1	
		G/G-G/C	120	305	0.96 (0.68–1.36)	0.818
	Recessive	G/C-C/C	177	437	1	
		G/G	23	71	0.81 (0.48–1.37)	0.440
	Log-additive	–	–	–	0.93 (0.72–1.20)	0.580

*SNP* Single nucleotide polymorphism, *CI* Confidence interval, *OR* Odds ratio

*indicates statistical significance (*p* < 0.05)

*P*^*a*^-values were calculated by unconditional logistic regression analysis without adjustment for age and gender

*P*^*b*^-values were calculated by unconditional logistic regression analysis with adjustment for age and gender

### Stratification analyses

To further investigate the effect of confounding factors on the association of *MIR31HG* variants with SONFH occurrence, we conducted a stratified analysis based on age, gender, disease course, and bilateral. The age-stratified analysis of the relationship between SNPs and SONFH risk is presented in Table 4. In subjects aged > 40 years, rs10965059 was related to a reduced risk of SONFH (T allele: OR = 0.30, *p* < 0.001; C/T genotype: OR = 0.34, *p* < 0.001; C/T-T/T genotype: OR = 0.33, *p* < 0.001). Conversely, no significant relationship of *MIR31HG* variants with SONFH risk was observed in subjects aged less than 40 years.

**Table 4:** Correlation between *MIR31HG* SNPs and SONFH risk stratified by age

SNP	Allele/Genotype	Case	Control	OR (95% CI)	*p*	Case	Control	OR (95% CI)	*p*
Age		> 40				≤40			
rs1332184	G	149	426	1		153	320	1	
	A	51	152	0.96 (0.67–1.39)	0.825	47	116	0.85 (0.58–1.25)	0.413
	G/G	53	154	1		56	122	1	
	G/A	43	118	1.02 (0.62–1.68)	0.937	41	76	1.18 (0.72–1.93)	0.522
	A/A	4	17	0.70 (0.22–2.31)	0.564	3	20	0.33 (0.09–1.15)	0.080
	G/A-A/A	47	135	0.98 (0.61–1.59)	0.939	44	96	1.00 (0.62–1.61)	0.995
rs72703442	C	169	481	1		174	368	1	
	A	31	99	0.89 (0.57–1.38)	0.608	26	66	0.83 (0.51–1.36)	0.466
	C/C	70	195	1		75	157	1	
	C/A	29	91	0.79 (0.47–1.34)	0.386	24	54	0.93 (0.53–1.62)	0.799
	A/A	1	4	0.70 (0.07–6.83)	0.764	1	6	0.35 (0.04–2.95)	0.334
	C/A-A/A	30	95	0.79 (0.47–1.33)	0.371	25	60	0.87 (0.51–1.50)	0.621
rs2025327	T	176	511	1		181	381	1	
	C	24	69	1.01 (0.62–1.66)	0.969	19	55	0.73 (0.42–1.26)	0.255
	T/T	78	224	1		81	167	1	
	T/C	20	63	0.86 (0.47–1.56)	0.618	19	47	0.83 (0.46–1.51)	0.549
	C/C	2	3	1.16 (0.17–7.90)	0.882	0	4	/	0.999
	T/C-C/C	22	66	0.88 (0.49–1.56)	0.657	19	51	0.77 (0.43–1.39)	0.381
rs55683539	C	155	429	1		153	338	1	
	T	45	151	0.82 (0.56–1.21)	0.320	47	96	1.08 (0.73–1.59)	0.706
	C/C	58	162	1		58	135	1	
	C/T	39	105	1.07 (0.65–1.76)	0.790	37	68	1.27 (0.76–2.10)	0.359
	T/T	3	23	0.32 (0.09–1.17)	0.085	5	14	0.83 (0.29–2.42)	0.734
	C/T-T/T	42	128	0.93 (0.57–1.50)	0.753	42	82	1.19 (0.74–1.93)	0.475
rs2181559	T	132	362	1		134	289	1	
	A	68	218	0.86 (0.61–1.20)	0.364	64	147	0.94 (0.67–1.33)	0.739
	T/T	41	113	1		46	100	1	
	T/A	50	136	1.08 (0.65–1.81)	0.746	42	89	1.03 (0.62–1.70)	0.921
	A/A	9	41	0.49 (0.21–1.16)	0.105	11	29	0.83 (0.38–1.79)	0.627
	T/A-A/A	59	177	0.93 (0.57–1.52)	0.774	53	118	0.98 (0.61–1.57)	0.922
rs10965059	C	186	451	1		173	378	1	
	T	14	113	0.30 (0.17–0.54)	< 0.001*	27	56	1.05 (0.66–1.67)	0.845
	C/C	86	174	1		78	166	1	
	C/T	14	103	0.34 (0.18–0.65)	< 0.001*	17	46	0.79 (0.42–1.46)	0.446
	T/T	0	5	/	0.999	5	5	2.13 (0.60–7.57)	0.243
	C/T-T/T	14	108	0.33 (0.18–0.62)	< 0.001*	22	51	0.92 (0.52–1.62)	0.768
rs10965064	C	126	350	1		131	290	1	
	G	74	230	0.89 (0.64–1.25)	0.507	69	146	1.05 (0.74–1.47)	0.807
	C/C	37	103	1		43	100	1	
	C/G	52	144	0.96 (0.57–1.62)	0.884	45	90	1.16 (0.70–1.93)	0.559
	G/G	11	43	0.67 (0.30–1.50)	0.334	12	28	1.00 (0.46–2.14)	0.993
	C/G-G/G	63	187	0.90 (0.55–1.47)	0.662	57	118	1.12 (0.70–1.81)	0.633

*SNP* Single nucleotide polymorphism, *CI* Confidence interval, *OR* Odds ratio

*P* **-**Values were calculated by logistic regression adjusted by age and gender

*indicates statistical strongly significance (*p* < 0.01)

Gender-based stratified analysis (Table 5) indicated that, rs10965059 was a protective SNP for SONFH in males (T allele: OR = 0.53, *p* = 0.009; C/T genotype: OR = 0.35, *p* < 0.001; C/T-T/T genotype: OR = 0.42, *p* = 0.001).

**Table 5:** Correlation between SNPs and SONFH susceptibility stratified by gender

SNP	Allele/Genotype	Case	Control	OR (95% CI)	*p*	Case	Control	OR (95% CI)	*p*
Gender		Male				Female			
rs1332184	G	174	630	1		128	116	1	
	A	60	220	0.98 (0.70–1.37)	0.904	38	48	0.72 (0.44–1.18)	0.187
	G/G	60	238	1		49	38	1	
	G/A	54	154	1.39 (0.91–2.13)	0.123	30	40	0.59 (0.31–1.11)	0.100
	A/A	3	33	0.35 (0.10–1.18)	0.089	4	4	0.77 (0.18–3.30)	0.729
	G/A-A/A	57	187	1.21 (0.80–1.82)	0.375	34	44	0.60 (0.33–1.12)	0.108
rs72703442	C	202	712	1		141	137	1	
	A	32	136	0.82 (0.54–1.26)	0.363	25	29	0.84 (0.47–1.50)	0.552
	C/C	85	298	1		60	54	1	
	C/A	32	116	0.97 (0.61–1.54)	0.891	21	29	0.65 (0.33–1.27)	0.209
	A/A	0	10	/		2	0	/	0.999
	C/A-A/A	32	126	0.89 (0.56–1.41)	0.618	23	29	0.71 (0.37–1.38)	0.310
rs2025327	T	207	753	1		150	139	1	
	C	27	97	0.99 (0.63–1.57)	0.975	16	27	0.55 (0.28–1.06)	0.072
	T/T	91	333	1		68	58	1	
	T/C	25	87	1.04 (0.63–1.71)	0.892	14	23	0.52 (0.24–1.10)	0.085
	C/C	1	5	0.68 (0.08–5.92)	0.725	1	2	0.40 (0.03–4.63)	0.463
	T/C-C/C	26	92	1.02 (0.62–1.67)	0.952	15	25	0.51 (0.24–1.05)	0.069
rs55683539	C	182	640	1		126	127	1	
	T	52	208	0.90 (0.64–1.26)	0.535	40	39	1.03 (0.62–1.71)	0.898
	C/C	69	248	1		47	49	1	
	C/T	44	144	1.13 (0.73–1.74)	0.587	32	29	1.16 (0.61–2.20)	0.658
	T/T	4	32	0.46 (0.16–1.34)	0.152	4	5	0.83 (0.21–3.27)	0.785
	C/T-T/T	48	176	1.00 (0.66–1.53)	0.986	36	34	1.11 (0.60–2.05)	0.747
rs2181559	T	155	551	1		111	100	1	
	A	79	299	0.95 (0.70–1.29)	0.725	53	66	0.72 (0.46–1.14)	0.159
	T/T	46	183	1		41	30	1	
	T/A	63	185	1.42 (0.92–2.19)	0.117	29	40	0.53 (0.27–1.04)	0.064
	A/A	8	57	0.55 (0.24–1.24)	0.149	12	13	0.67 (0.27–1.68)	0.392
	T/A-A/A	71	242	1.20 (0.79–1.83)	0.390	41	53	0.56 (0.30–1.05)	0.072
rs10965059	C	210	691	1		149	138	1	
	T	24	153	0.53 (0.33–0.85)	0.009*	17	16	0.98 (0.48–2.02)	0.965
	C/C	97	277	1		67	63	1	
	C/T	16	137	0.35 (0.20–0.63)	< 0.001*	15	12	1.17 (0.51–2.70)	0.709
	T/T	4	8	1.45 (0.42–4.94)	0.555	1	2	0.49 (0.04–5.54)	0.561
	C/T-T/T	20	145	0.42 (0.25–0.71)	0.001*	16	14	1.08 (0.49–2.39)	0.857
rs10965064	C	156	536	1		101	104	1	
	G	78	314	0.87 (0.64–1.18)	0.370	65	62	1.08 (0.69–1.68)	0.735
	C/C	52	171	1		28	32	1	
	C/G	52	194	0.91 (0.59–1.41)	0.669	45	40	1.28 (0.66–2.49)	0.461
	G/G	13	60	0.73 (0.37–1.44)	0.359	10	11	1.03 (0.38–2.80)	0.958
	C/G-G/G	65	254	0.87 (0.57–1.31)	0.496	55	51	1.23 (0.65–2.32	0.526

*SNP* Single nucleotide polymorphism, *CI* Confidence interval, *OR* Odds ratio

*p* values were calculated by logistic regression adjusted by age and gender

*indicates statistical strongly significant (*p* < 0.01)

Additionally, the stratification analysis of the association between *MIR31HG* SNPs and SONFH risk by course and bilateral are presented in Table 6. We discovered that rs2025327 might contribute to prolonged SONFH course (OR = 2.14, *p* = 0.046). The rs10965059 was associated with the reduced risk of bilateral SONFH (T allele, OR = 0.56, *p* = 0.005; C/T genotype, OR = 0.43, *p* = 0.002; C/T-T/T genotype, OR = 0.51, *p* = 0.007).

**Table 6:** Relationships of *MIR31HG* SNPs with SONFH risk stratified by course and bilateral

SNP	Allele/Genotype	Case 1 (course > 29 month)	Case 2 (course ≤29 month)	OR (95% CI)	*p*	Cases with bilateral	Control	OR (95% CI)	*p*
rs1332184	G	95	207	1		216	746	1	
	A	27	71	0.83 (0.50–1.37)	0.466	70	268	0.90 (0.67–1.22)	0.506
	G/G	35	74	1		77	276	1	
	G/A	25	59	0.10 (0.53–1.88)	0.987	62	194	1.10 (0.74–1.63)	0.650
	A/A	1	6	0.27 (0.03–2.45)	0.246	4	37	0.38 (0.13–1.14)	0.084
	G/A-A/A	26	65	0.91 (0.49–1.70)	0.764	66	231	0.98 (0.67–1.45)	0.935
rs72703442	C	106	237	1		244	849	1	
	A	16	41	0.87 (0.47–1.62)	0.667	42	165	0.89 (0.61–1.28)	0.517
	C/C	45	100	1		102	352	1	
	C/A	16	37	0.98 (0.48–1.97)	0.949	40	145	0.93 (0.60–1.43)	0.727
	A/A	0	2	/		1	10	0.40 (0.05–3.27)	0.393
	C/A-A/A	16	39	0.90 (0.45–1.81)	0.767	41	155	0.90 (0.59–1.37)	0.610
rs2025327	T	105	252	1		259	892	1	
	C	17	26	1.57 (0.82–3.01)	0.173	27	124	0.75 (0.48–1.16)	0.197
	T/T	44	115	1		117	391	1	
	T/C	17	22	2.14 (1.01–4.53)	0.046*	25	110	0.70 (0.43–1.16)	0.164
	C/C	0	2	/		1	7	0.49 (0.06–4.09)	0.506
	T/C-C/C	17	24	1.93 (0.92–4.02)	0.081	26	117	0.69 (0.42–1.13)	0.136
rs55683539	C	92	216	1		220	767	1	
	T	30	62	1.14 (0.69–1.87)	0.617	66	247	0.93 (0.68–1.27)	0.654
	C/C	33	83	1		82	297	1	
	C/T	26	50	1.35 (0.71–2.57)	0.355	56	173	1.16 (0.77–1.73)	0.481
	T/T	2	6	0.80 (0.15–4.37)	0.796	5	37	0.52 (0.19–1.41)	0.198
	C/T-T/T	28	56	1.29 (0.69–2.41)	0.422	61	210	1.05 (0.71–1.55)	0.812
rs2181559	T	75	191	1		193	651	1	
	A	45	87	1.37 (0.84–2.06)	0.228	91	365	0.84 (0.64–1.11)	0.225
	T/T	22	65	1		63	213	1	
	T/A	31	61	1.68 (0.85–3.31)	0.135	67	225	1.04 (0.69–1.56)	0.854
	A/A	7	13	1.54 (0.53–4.48)	0.427	12	70	0.53 (0.27–1.07)	0.078
	T/A-A/A	38	74	1.65 (0.87–3.15)	0.128	79	295	0.91 (0.62–1.35)	0.640
rs10965059	C	108	251	1		257	829	1	
	T	14	27	1.21 (0.61–2.39)	0.592	29	169	0.56 (0.36–0.84)	0.005*
	C/C	48	116	1		119	340	1	
	C/T	12	19	1.54 (0.68–3.50)	0.301	19	149	0.43 (0.25–0.74)	0.002*
	T/T	1	4	0.87 (0.09–8.29)	0.900	5	10	1.55 (0.49–4.89)	0.452
	C/T-T/T	13	23	1.45 (0.66–3.16)	0.357	24	159	0.51 (0.31–0.83)	0.007*
rs10965064	C	78	179	1		184	640	1	
	G	44	99	1.02 (0.66–1.59)	0.931	102	376	0.94 (0.72–1.24)	0.677
	C/C	21	59	1		55	203	1	
	C/G	36	61	1.53 (0.79–2.97)	0.212	74	234	1.11 (0.76–1.68)	0.612
	G/G	4	19	0.52 (0.15–1.76)	0.290	14	71	0.73 (0.37–1.43)	0.359
	C/G-G/G	40	80	1.29 (0.68–2.45)	0.440	88	305	1.03 (0.69–1.53)	0.896

*SNP* Single nucleotide polymorphism, *CI* Confidence interval, *OR* Odds ratio

*p* values were calculated by logistic regression adjusted by age and gender

*indicates statistical significance (*p* < 0.05)

### MDR analysis of SNP-SNP interaction on SONFH

**Fig. 1:**
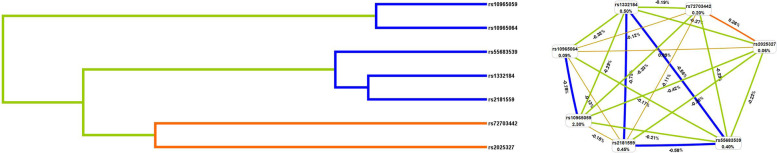
The tree diagram analysis among SNP interaction

SNP-SNP interaction was determined using MDR analysis. As shown in Table 7 and Fig. 1, the analysis results indicated that the combination of rs1332184, rs72703442, rs2025327, rs55683539, rs2181559, rs10965059, and rs10965064 was the optimal model for predicting SONFH occurrence (training accuracy = 0.671, CVC = 10/10, *p* < 0.0001). In addition, the optimal single locus model for predicting SONFH risk was rs10965059 (training accuracy = 0.578, CVC = 10/10, *p* < 0.0001). Two-locus model was rs2181559 and 10,965,059. Three-locus model was consisted of rs2025327, rs2181559, and rs10965059. Four--locus model was consisted of rs1332184, rs2181559, rs10965059 and rs10965064. Five--locus model was the combination of rs1332184, rs72703442, rs55683539, rs2181559, rs10965059 and rs10965064. The results of the network diagram and the tree diagram were consistent (Fig. 1). There was a stronger redundant interaction between rs10965059 and rs10965064 (information gain: − 0.78%) and a stronger synergy between rs72703442 and rs2025327 (information gain: 0.26%).

**Table 7:** Analysis of SNP-SNP interaction models using MDR method

Model	Training Bal. Acc.	Testing Bal. Acc.	CVC	OR (95% CI)	*p*
rs10965059	0.578	0.574	10/10	2.46 (1.61–3.77)	<0.0001
rs2181559, rs10965059	0.598	0.577	7/10	2.60 (1.76–3.83)	<0.0001
rs2025327, rs2181559, rs10965059	0.609	0.555	5/10	3.07 (2.03–4.65)	<0.0001
rs1332184, rs2181559, rs10965059, rs10965064	0.634	0.523	7/10	2.93 (2.07–4.16)	<0.0001
rs1332184, rs72703442, rs2181559, rs10965059, rs10965064	0.652	0.521	7/10	3.34 (2.37–4.72)	<0.0001
rs1332184, rs72703442, rs55683539, rs2181559, rs10965059, rs10965064	0.666	0.542	7/10	3.79 (2.68–5.36)	<0.0001
rs1332184, rs72703442, rs2025327, rs55683539, rs2181559, rs10965059, rs10965064	0.671	0.543	10/10	4.39 (3.01–6.40)	<0.0001

*Bal. Acc *Balanced accuracy, *CVC *Cross-validation consistently, *CI *Confidence interval, *OR *Odds ratio

*p* values were calculated by χ^2^ test

*p* < 0.01 indicates statistical strongly significant

### The correlation of *MIR31HG* haplotypes with SONFH susceptibility

**Fig. 2:**
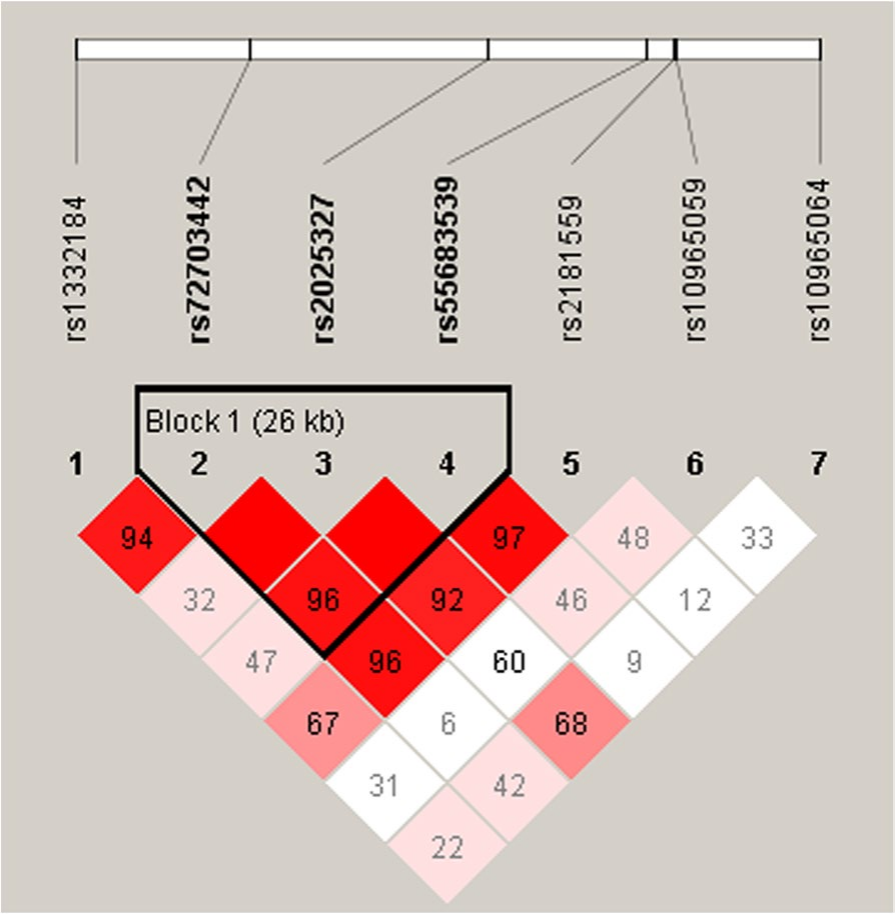
The linkage disequilibrium structure of seven SNPs in the *MIR31HG* gene. The numbers in squares are D′ values

We also examined the impacts of *MIR31HG* haplotypes on SONFH susceptibility. As shown in Fig. 2, a linkage disequilibrium (LD) block was comprised of three SNPs including rs72703442, rs2025327 and rs55683539. The frequency distribution of haplotypes in case and control group is presented in Table 8. To examine the effect of haplotypes on SONFH risk, a haplotype-based logistic regression method was carried out in the case–control cohort, however, no significant association was found.

**Table 8:** Relationships of *MIR31HG* haplotypes with SONFH risk

Blocks	SNPs	Haplotype	Frequency		Crude analysis		Adjusted by age and gender	
			Case	Control	OR (95% CI)	*p*	OR (95% CI)	*p*
Block 1	rs72703442|rs2025327|rs55683539	ATT	0.138	0.159	0.84 (0.60–1.18)	0.305	0.82 (0.58–1.17)	0.271
	rs72703442|rs2025327|rs55683539	CTT	0.093	0.084	1.11 (0.75–1.65)	0.607	1.16 (0.76–1.75)	0.497
	rs72703442|rs2025327|rs55683539	CCC	0.108	0.122	0.86 (0.60–1.25)	0.435	0.82 (0.56–1.21)	0.319
	rs72703442|rs2025327|rs55683539	CTC	0.343	0.369	0.89 (0.70–1.14)	0.360	0.88 (0.68–1.12)	0.300

*SNP* Single nucleotide polymorphism, *CI* Confidence interval, *OR* Odds ratio

*p* values were calculated by logistic regression adjusted by age and gender

## Discussion

SONFH is multi-layered and intricate disease with femoral neck fracture or bone tissue disorder, whose symptoms and signs are diverse, and the time and degree of pain attack are different However, SONFH still has the basis of pathological evolution. There are no specific clinical manifestations of ONFH, so it is difficult to make a diagnosis of ONFH from the patient’s symptoms and clinical examination [16]. With the development of modern precision medicine in recent years, an in-depth research on stem cells, molecular biology, and the exact pathogenesis of SONFH has been analyzed. A large number of experiments have shown that the increase in reactive oxygen species (ROS) caused by hormone use is related to the occurrence and development of SONFH [17, 18]. The the frequent collapse of the femoral head and hip joint dysfunction makes the treatment of SONFH difficult [4, 19].

LncRNA consists of a non-protein coding transcripts with approximately 200 nucleotides [6], which are drawn into various cellular processes such as chromatin remodeling, post-transcriptional processing and transcription process [20], involved in the occurrence, progression, and metastasis of human cancers, and played corresponding roles. Among those cancers, lncRNAs are more widely researched in osteosarcoma, including *lncRNA-21A*, *UCA1*, *MEG3*, *HULC,* and *MIR31HG* [21]. *MIR31HG* acts as an oncogene in osteosarcoma to promote tumor progression via regulation of tumor suppressor miR-361 and its target genes [9, 21]. Taken together, studying SONFH in the field of exploring lncRNAs is highly needed and promising. Moreover, according to our current research results, there is a significant correlation between *MIR31HG* polymorphism and SONFH susceptibility in the Chinese Han population.

*MIR31HG* is a kind of lncRNA that can be expressed in human bone cells, and it involves autoimmune in the recent research reports. The most noteworthy thing is that there is no research on the correlation between *MIR31HG* polymorphism and SONFH susceptibility. Our research is the first to find a significant risk connection between *MIR31HG* genetic variations and SONFH susceptibility in the Han population in China. The locus in *MIR31HG* has only been reported in IgAN currently [8]. In future studies, if SNP assessment is used as a type of risk marker, patients with high risk of ONFH can be identified through screening, and the dosage of steroids then can be differentiated based on individual differences, which can prevent the development of SONFH [1].

Therefore, we are committed to investigating the association between the *MIR31HG* gene polymorphism and the risk of SONFH disease. Our study results of genotyping showed that rs10965059-T allele frequency in the case group (0.103) was lower than that in the control group (0.169), and the reduced risk of SONFH was found. The stratified analysis results showed that rs10965059 was associated with the reduced risk of SONFH in subjects aged > 40 years (*p* < 0.001), and males (*p* < 0 .001). Consequently, we speculated that age and gender may interact with *MIR31HG* genetic polymorphisms on SONFH occurrence. Moreover, rs10965059 was associated with the reduced risk of bilateral SONFH (*p* = 0.002).

However, there are other candidate genes in the research on SONFH, and the research on *MIR31HG* is relatively rare. Nonetheless, our current work has some limitations. First of all, the relationship between SNPs and SONFH risk was investigated in the early stage, and the relationship among gene-environment interactions needs to be studied in the later work. Second, we have successfully demonstrated the relationship between *MIR31HG* polymorphisms and SONFH, and the molecular mechanism of SONFH will be studied in the future work. Patients were all from Shandong, Inner Mongolia and adjacent areas, which are in low population mobility. It is easy to carry out population-based research. As is known to all, this is the first study to probe into the effect of *MIR31HG* mutation on SONFH, which may provide a scientific basis for future research of *MIR31HG* on the molecular mechanism of SONFH.

## Conclusion

Our study indicates that rs10965059 in *MIR31HG* is a protective SNP for SONFH, which offers a new insight for the molecular mechanism and provides a new major candidate gene in the study progression of SONFH. In the future, we will continue to collect samples to expand the sample size for confirming our results in a larger cohort of subjects.

## Supplementary Information

**Additional file 1: Supplemental Table 1.** Primers used for this study

## Data Availability

The datasets generated and/or analysed during the current study are not publicly available due [REASON WHY DATA ARE NOT PUBLIC] but are available from the corresponding author on reasonable request.
