# Supplementary material for: MIR31HG polymorphisms are related to steroid-induced osteonecrosis of femoral head among Chinese Han population
Source: BMC Musculoskelet Disord. 2022 Sep 3;23:836. doi: 10.1186/s12891-022-05785-w (PMC9440494; doi:10.1186/s12891-022-05785-w)
Supplement: Supplementary file 1 — Additional file 1: Supplemental Table 1. Primers used for this study [file 12891_2022_5785_MOESM1_ESM.docx]

**Supplemental Table 1** Primers used for this study

| **SNP** | **1st-PCRP** | **2nd-PCRP** | **UEP_DIR** | **UEP_SEQ** |
| --- | --- | --- | --- | --- |
| rs1332184 | ACGTTGGATGGCTGCAGTTGCTTTCAATAA | ACGTTGGATGAAAGCACTGCTGGAAAGCCT | F | atgaTGGAAAGCCTGAGCAGAGAAAT |
| rs72703442 | ACGTTGGATGGTCTTTGGTGGTGTTCTTCC | ACGTTGGATGAGGCAGCTTATAGGAATGGC | F | gggtAAGTGTCTTTACATTAGCAAGC |
| rs2025327 | ACGTTGGATGATCCAAAATCTCAGCGGTAG | ACGTTGGATGTAGTGTGTGACCTACATCTG | F | TGACCTACATCTGAGTTTC |
| rs55683539 | ACGTTGGATGTGGATCATCAGCAGTCCAG | ACGTTGGATGTCAGCCAGTTTAGCCACACT | R | TGAGAAAACAGTCATATCCT |
| rs2181559 | ACGTTGGATGCGTGAGACCTATTCACAAGC | ACGTTGGATGGTTATGGGAAGAACTTGGTG | F | gggGAACTTGGTGGGAGGT |
| rs10965059 | ACGTTGGATGTGGCAGAAGGTGAAAGGCAA | ACGTTGGATGTCTCACGGTTTTAAGAAGGG | F | gTCTTGTCTGCTGCCA |
| rs10965064 | ACGTTGGATGGTAGTACTGGGACTCCAATC | ACGTTGGATGGAGTTCCACAGATATTCTGC | F | AAGAGCAGAGACATAGAAATAG |

SNP, single nucleotide polymorphism.
